# Effect of *Ganoderma lucidum* spores intervention on glucose and lipid metabolism gene expression profiles in type 2 diabetic rats

**DOI:** 10.1186/s12944-015-0045-y

**Published:** 2015-05-22

**Authors:** Fang Wang, Zhongkai Zhou, Xiaochong Ren, Yuyang Wang, Rui Yang, Jinhua Luo, Padraig Strappe

**Affiliations:** Key Laboratory of Food Nutrition and Safety, Ministry of Education, Tianjin University of Science and Technology, Tianjin, 300457 China; Chongqing Biotechnology Research Institute, Chongqing, 401121 China; School of Biomedical Sciences, Charles Sturt University, Wagga Wagga, NSW 2678 Australia; School of Food Engineering and Biotechnology, Tianjin University of Science and Technology, Tianjin, 300457 China

**Keywords:** *Ganoderma lucidum*, Blood glucose, Lipid composition, Gene expression, Type 2 diabetes

## Abstract

**Background:**

The fruiting body of *Ganoderma lucidum* has been used as a traditional herbal medicine for many years. However, to the date, there is no detailed study for describing the effect of *G. lucidum* spores on oxidative stress, blood glucose level and lipid compositions in animal models of type 2 diabetic rats, in particular the effect on the gene expression profiles associated with glucose and lipid metabolisms.

**Methods:**

*G. lucidum* spores powder (GLSP) with a shell-broken rate >99.9 % was used. Adult male Sprague–Dawley rats were randomly divided into three groups (n = 8/group). Group 1: Normal control, normal rats with ordinary feed; Group 2: Model control, diabetic rats with ordinary feed without intervention; Group 3: GLSP, diabetic rats with ordinary feed, an intervention group utilizing GLSP of 1 g per day by oral gavages for 4 consecutive weeks. Type 2 diabetic rats were obtained by streptozocin (STZ) injection. The changes in the levels of glucose, triglycerides, total cholesterol and HDL-cholesterol in blood samples were analyzed after GLSP intervention. Meanwhile, gene expressions associated with the possible molecular mechanism of GLSP regulation were also investigated using a quantitative RT-PCR.

**Results:**

The reduction of blood glucose level occurred within the first 2 weeks of GLSP intervention and the lipid synthesis in the diabetic rats of GLSP group was significantly decreased at 4 weeks compared to the model control group. Furthermore, it was also found that GLSP intervention greatly attenuated the level of oxidative stress in the diabetic rats. Quantitative RT-PCR analysis showed up-regulation of lipid metabolism related genes (*Acox1*, *ACC*, *Insig-1* and *Insig-2*) and glycogen synthesis related genes (*GS2* and *GYG1*) in GLSP group compared to model control group. Additionally, there were no significant changes in the expression of other genes, such as *SREBP-1, Acly, Fas, Fads1, Gpam, Dgat1, PEPCK* and *G6PC1*.

**Conclusion:**

This study might indicate that GLSP consumption could provide a beneficial effect in terms of lowering the blood glucose levels by promoting glycogen synthesis and inhibiting gluconeogenesis. Meanwhile, GLSP treatment was also associated with the improvement of blood lipid compositions through the regulation of cholesterol homeostasis in the type 2 diabetic rats.

**Electronic supplementary material:**

The online version of this article (doi:10.1186/s12944-015-0045-y) contains supplementary material, which is available to authorized users.

## Introduction

Diabetes mellitus (DM) is a metabolic disorder caused by a lack of insulin and/or pancreatic dysfunction characterized by hyperglycemia. DM is a common, morbid and costly disease, affecting more than 1 in 10 adults in both United States and China [1]. DM is the leading cause of new blindness, amputation and end-stage renal diseases and it also contributes to a host of other conditions. Although there is no published data for the medical cost of diabetes in China in recent years, the medical costs of managing DM and its complications exceeded $115 billion in the United States alone in 2011 and the indirect costs added another $58 billion [1]. Evidence suggests that DM complications can be markedly attenuated with appropriate control of blood pressure and hyperglycemia and with successful treatment of hyperlipidemia. Thus, there is a great interest in novel approaches to indirect DM management. However, despite the increasing number of drugs available for DM treatment, significant improvements in the control of DM have not been observed [2].

Previous studies have confirmed that the treatment with natural antioxidants can reduce diabetic complications [3] and the continuous efforts to discover new antioxidants as useful drug candidates to combat diabetic complications are on-going. *Ganoderma lucidum* (Leyss; Fr) Karst. (*Ganodermataceae*) is a well-known Chinese traditional medicine which has been clinically used in China, Japan and Korea for more than 2000 years. Mushrooms of the genus *Ganoderma* have been shown to be a rich source of biologically active metabolites [4], containing many bioactive components, including triterpenoids, polysaccharides, nucleotides, sterols, steroids, peptides and other bioactive ingredients [5]. *G. lucidum* spores contain high levels of ganoderic acids, ergosterol peroxide and pentadecanoate [6]. Many are active against current major chronic diseases. For example, ganoderic acids, one group of triterpenoids existing in the fruiting body of *G. lucidum* showed anti-androgenic, anti-5 α-reductase, anti-inflammatory and anti-tumor and a range of other biological activities [7–9].

Although the fruiting body of *G. lucidum* has been used as a traditional herbal medicine since ancient times, the spores were utilized only in the late 20th century [10]. The spores contain many bioactive substances, including lanostane type triterpenes [11] and polysaccharides [12] similar to those in the fruiting body [13]. Other characteristics of the bioactive compounds existing in the spores are those they are also rich in fatty acids, in particular long-chain C-19 fatty acids. Previous study demonstrated that these fatty acids could inhibit tumor cell proliferation and induce apoptosis in the HL-60, promyelocytic leukemic cell line [14]. Meanwhile, other research also showed the potential anti-hyperglycemic effect in diabetic rats using polysaccharides extracted from *G. lucidum* fruiting body [15]. However, to the date, few detailed studies described the effects of *G. lucidum* spores on blood glucose and lipid compositions in streptozotocin (STZ) induced diabetic rats and neither of the investigation of *G. lucidum* spores intervention on the gene expression of glucose and lipid metabolisms has been reported in above diabetic model. To the best of our knowledge, there are few reports in the literature evaluating the feasibility of using *G. lucidum* spores as a potential anti-diabetic agent and the descriptions of the molecular mechanism(s) involved in these processes are also very rare. Moreover, the co-existing of the multi-active compounds in *G. lucidum* spores might provide a stronger synergistic or positively effect on improving the diabetic status than the consumption of the single active compound. Therefore, in this study, STZ-induced-diabetic rats are used to investigate the changes in the expression levels of genes involved in lipid and glucose metabolisms after *G. lucidum* spores treatment.

## Results

### Effect of GLSP intervention on the body mass of diabetic rats

There was no significant difference in the initial weights among the three groups (*P* = 0.6925) (Additional file 1: Table S1), indicating that the random grouping was acceptable. The body mass of the rats in the normal control group gradually increased at 4 weeks, suggesting that the dietary composition designed was appropriate. After the injection of STZ, the rats in the two diabetic groups (*i.e.* model control group and GLSP intervention group) displayed a reduction of body mass gain and the weight of the diabetic rats was lower than that of the normal control group (*P* > 0.01) during the 4 consecutive weeks, suggesting that the diabetic disease greatly affected the normal development of these rats. There was no significant difference in the body mass between the model control and GLSP intervention group (*P* = 0.6099) within the first 3 weeks. At the fourth week, the body mass of GLSP group was higher by 9.0 % (*P* > 0.05) compared to that of model group, indicating that GLSP consumption could partially recover the weight loss after 4 weeks treatment.

### Effect of GLSP intervention on blood glucose and insulin levels in the diabetic rats

Following the injection of STZ, these animals displayed the expected symptoms of insulin-dependent diabetes mellitus, *i.e.* hyperglycemia, with glucose level around 30 mmol/L within 72 h of STZ injection. This symptom remained relatively constant during the 4 subsequent weeks. As shown in Table 1, GLSP intervention for 4 weeks led to a 21.0 % reduction of blood glucose level compared to its corresponding initial level. Moreover, the blood glucose level of the rats in GLSP group was significantly lower than that of rats in model control group (*P* < 0.05). Rats of GLSP intervention at 4 weeks also exhibited a significant increase in the insulin level compared to the rats of model control group (*P* < 0.05, unpublished data).

**Table 1:** Effect of GLSP intervention on blood glucose level

Blood glucose level (mmol/L)						RMANOVA *F* value	*P* value
Group	72 h	1 week	2 week	3 week	4 week		
Normal control	5.96 ± 0.64^b^	5.80 ± 0.83^b^	5.72 ± 0.29^b^	5.82 ± 0.79^b^	6.20 ± 0.52^c^	0.633	0.646
Model control	32.28 ± 1.00^a^	30.00 ± 3.32^a^	24.12 ± 6.60^a^	30.96 ± 3.04^a^	32.22 ± 1.71^a^	3.485	0.028
GLSP intervention	30.79 ± 2.72^a^	28.19 ± 8.32^a^	25.27 ± 3.98^a^	25.52 ± 7.48^a^	24.31 ± 1.17^b^	11.125	<0.001^**^

Results are expressed as means ± SD (*n* = 8, one-way ANOVA). Different superscript lowercase letters on the table indicate significant difference (*P* < 0.05). RMANOVA: Repeated measures ANOVA. **Strongly significant (*P* value: *P* < 0.001). Normal control: healthy rats without intervention; Model control: STZ induced diabetic rats without intervention; GLSP intervention: STZ induced diabetic rats with GLSP intervention

### Effect of GLSP intervention on blood lipid compositions

As shown in Table 2, diabetic rats in the model control group had a significant higher in blood triglyceride (TG) (*P* < 0.01) and total cholesterol (TC) (*P* < 0.01) compared to the normal rats. Following the therapeutic treatment of GLSP, the blood TG fell significantly by 49.0 % and TC reduced by 17.8 %, respectively, as compared to the non-treatment group (*i.e.* model control) (Table 2). Although GLSP intervention did not attenuate the level of TG and TC to a normal status, this study found that *G. lucidum* intervention could significantly enhance the level of high density lipoproteincholesterol (HDL-c) by 48.6 % (*P* < 0.01) compared to the model control. It is interesting to note that there was no significant difference in HDL-c between normal group and GLSP intervention group (*P* = 0.6850).

**Table 2:** Effect of GLSP intervention on blood TG, TC and HDL-c in diabetic rats

Group	TG (mmol/L)	TC (mmol/L)	HDL-c (mmol/L)
Normal control	0.29 ± 0.00^c^	2.96 ± 0.07^c^	2.90 ± 0.07^a^
Model control	2.92 ± 0.27^b^	5.57 ± 0.47^b^	1.32 ± 0.45^b^
GLSP intervention	1.49 ± 0.55^a^	4.58 ± 0.09^a^	2.57 ± 0.29^a^

Results are expressed as means ± SD (*n* = 8, one-way ANOVA). Different superscript lowercase letters on the table indicate significant difference (*P* < 0.05). *TG* triglyceride, *TC* total cholesterol, *HDL-c* high density lipoprotein cholesterol

### Effect of GLSP intervention on oxidative stress level

High level of blood glucose for a long term could induce mitochondrial reactive oxygen species (ROS). Thus, malondialdehyde (MDA) and ROS are the biomarkers commonly used for providing a reasonable index of oxidative stress status. Diabetic disease significantly increased the levels of oxidative stress (Table 3). For example, the blood MDA of diabetic rats without intervention (model control) increased by 42.3 % (*P* < 0.01) compared to normal ones and this was consistently accompanied by a significant increase in ROS as well (Table 3).

**Table 3:** Effect of GLSP intervention on the level of oxidative stress in diabetic rats

Group	MDA (nmol/mL)	ROS (U/mL)	GSH-Px (U/mg prot.)	SOD (U/mg prot.)
Normal control	6.49 ± 1.9^a^	53.96 ± 3.8^a^	1689.65 ± 220.1^a^	210.65 ± 7.7^b^
Model control	11.24 ± 0.2^b^	65.12 ± 7.6^a^	1223.26 ± 216.1^b^	179.26 ± 3.7^c^
GLSP intervention	9.68 ± 0.7^a^	61.12 ± 4.2^a^	1785.26 ± 259.7^a^	289.13 ± 4.0^a^

Results are expressed as means ± SD (*n* = 8, one-way ANOVA). Different superscript lowercase letters on the table indicate significant difference (*P* < 0.05). *MDA* malondialdehyde, *ROS* reactive oxygen species, *GSH-Px* glutathione peroxidase, *SOD* superoxide dismutase

As compared with the model control group, the MDA level was 13.9 % lower at 4 weeks in the GLSP group (Table 3) (*P* < 0.05). Furthermore, there was also a reduction of ROS for GLSP intervention group compared to the model control, although the difference was not significant. Nevertheless, the levels of glutathione peroxidase (GSH-Px) and superoxide dismutase (SOD) were higher by 25.9 % (*P* < 0.05) and 38.0 % (*P* < 0.05), respectively, following the GLSP treatment compared to the model control group.

### Expression of genes related to glucose metabolism

**Fig. 1:**
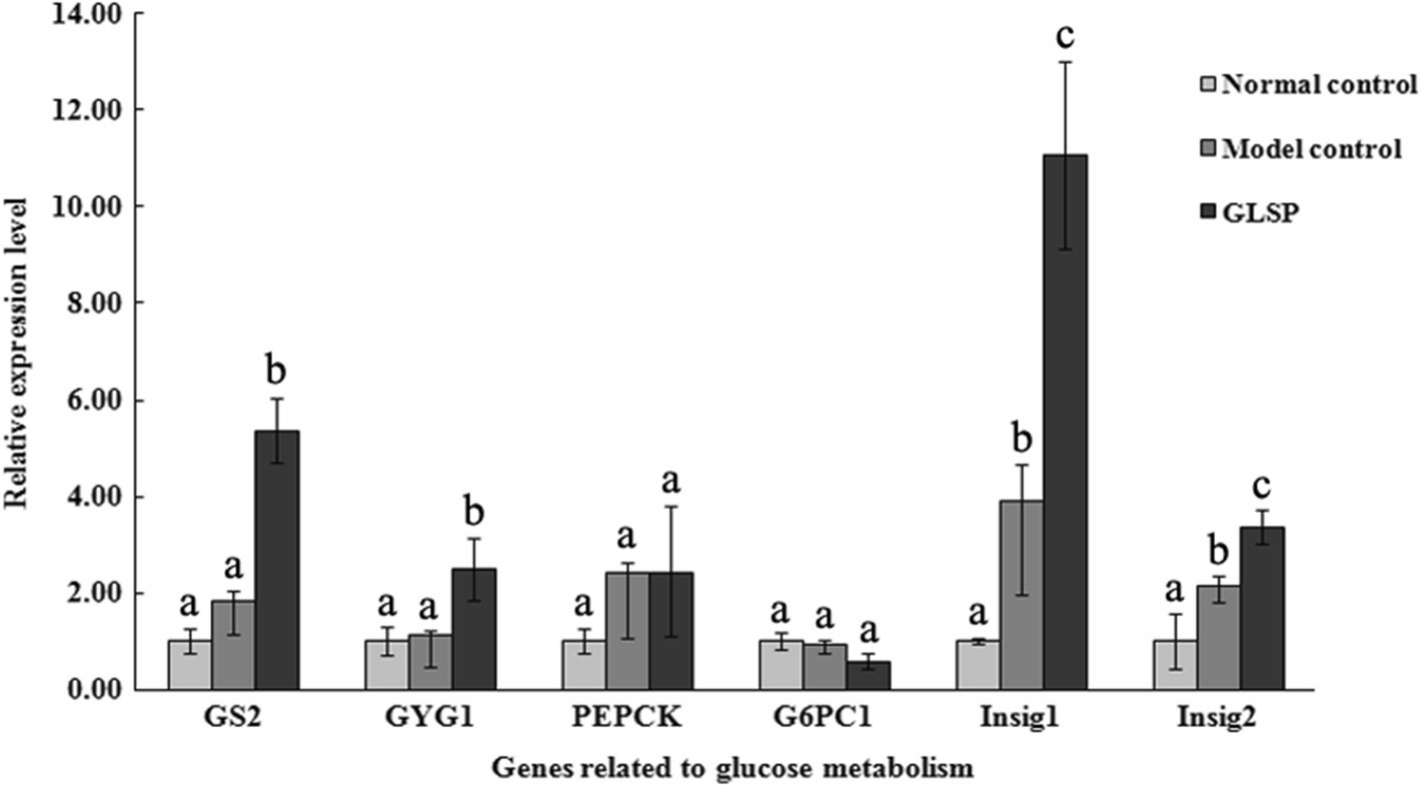
Hepatic gene expression profiles for glucose metabolism. Mean values with unlike letters are significantly different (one way ANOVA). Model control: STZ induced diabetic rats without intervention; Normal control: healthy rats without intervention; GLSP:STZ induced diabetic rats with GLSP intervention. *GS2* (glycogen synthase2, liver isoform) and *GYG1* (glycogenin 1): related to glycogen synthesis; *PEPCK* (phosphenolpyruvate carboxykinase) and *G6PC1* (isoform 1 of the catalytic subunit of glucose-6-phosphatase): related to gluconeogenesis; *Insig-1* (insulin induced gene 1) and *Insig-2* (insulin induced gene 2): responsible for glucose homeostasis

In order to understand the molecular mechanism associated with the reduction of blood glucose level following GLSP intervention, the expression of a number of genes involved in glucose metabolism in the liver was analyzed and the results are presented in Fig. 1. The analyses of genes encoding enzymes involved in glycogen synthesis, *i.e.* glycogen synthase2 (*GS2*) and glycogenin1 (*GYG1*) showed that, compared to the model control group, the expression level of hepatic *GS2*, the rate limiting enzyme of glycogen synthesis, was increased by 3-folds and *GYG1* also had a more than 2-folds increase at 4 weeks in the GLSP group. Furthermore, insulin-induced genes (*Insig-1* and *Insig-2*) were greatly up-regulated in the livers of GLSP treated type 2 diabetic rats. More importantly, the expression level of *isoform 1* of the catalytic subunit of glucose-6-phosphatase (*G6PC1*) was found to be greatly reduced after GLSP intervention, although there was no significant difference in the expression level of the genes related to gluconeogenesis, *i.e.*, phosphenolpyruvate carboxykinase (*PEPCK*) between GLSP intervention group and the model control group.

### Expression of genes related to lipids metabolism

**Fig. 2:**
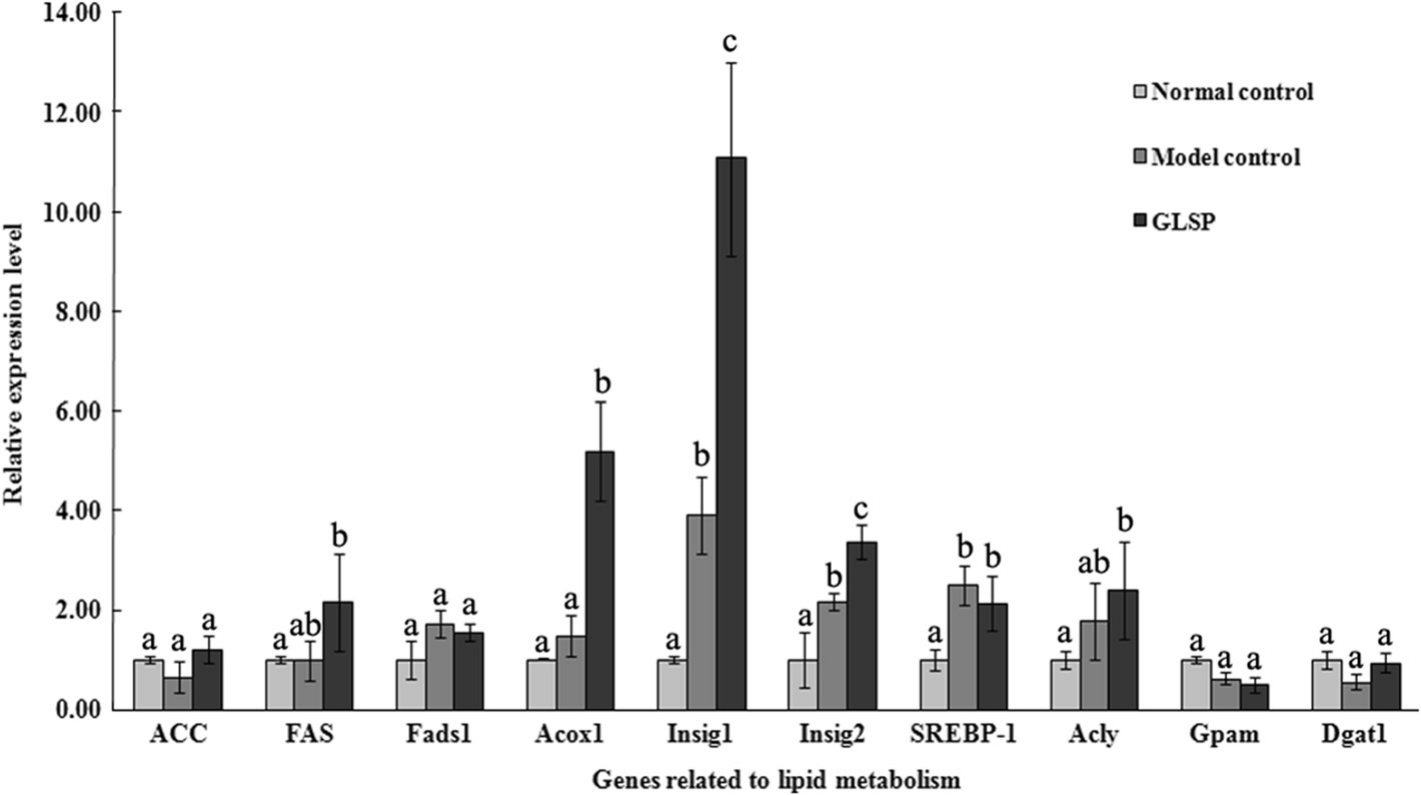
Hepatic gene expression profiles for lipid metabolism. Mean values with unlike letters are significantly different (one way ANOVA). *ACC* (Acetyl-CoA carboxylse) and *FAS* (fatty acid synthase): related to lipogenesis; *Gpam* (glycerol-3-phosphate acyltransferase mitochondrial) and *Dgat1* (diacylglycerol acyltransferase 1): responsible for triglycerides synthesis; *Fads1* (fatty acid desaturase 1): associated with fatty acid desaturation; *Acox1* (acyl-CoA oxidase 1): responsible for fatty acid oxidation; *Acly* (ATP citrate lyase): related to cholesterol biosynthesis; *Insig-1* (insulin induced gene 1) and *Insig-2* (insulin induced gene 2): regulation of cholesterol homeostasis; *SREBP-1* (sterol regulatory element binding protein-1): associated with the regulation of fatty acids and triglycerides syntheses and metabolism

The pathways involved in lipid metabolism are various, complexly inter-related to glucose and protein metabolisms [16]. To assess the changes associated with the control of lipogenesis, the expression level of gene encoding AcetylCoA carboxylase (ACC) was measured and there was no significant difference in the expression level among the three groups (Fig. 2). However, Acyl-CoA oxidase1 (*Acox1*), which is involved in lipid β-oxidation, was found to have the highest expression level in the type 2 diabetic rats following the GLSP intervention among the three groups. As described above, the expression levels of *Insig-1* and *Insig-2* were greatly up-regulated in the liver of GLSP treated type 2 diabetic rats compared to either normal rats or model control rats. Once more, there were no significant differences in the gene expression levels of *Fads*, *SREBP-1, Acyl, Gpam* and *Dgat1* between the model control group and GLSP intervention group (Fig. 2).

## Discussion

Although previous research has shown that the fruiting bodies of *G. lucidum* could reduce blood glucose and plasma cholesterol levels [17–19], few studies have investigated the molecular mechanisms associated with these activities. Our study combines the data of biochemical analysis and gene expression and indicates that GLSP intervention could result in a partial recovery of body weight, reduction of blood glucose level and improvement of lipid compositions and even attenuation of oxidative stress in the STZ-induced diabetic rats. These improvements might be highly associated with the changes in the expression levels of the related genes.

The current study would suggest that GLSP intervention could manipulate hyperglycemic and hyperlipidemic status with a significant reduction of blood glucose level and the improvement of blood lipid compositions (TG, TC, HDL-c) in type 2 diabetic rats (*P* < 0.01). The GLSP dosage used in this study was 1 g per day, which was about 3 % of the total diet. Although this dosage did not achieve a completely functional recovery of the DM rats to a normal status, it could be used as a reference dosage for improving the symptoms of DM. In contrast, Winther *et al.* [20] reported that *G. lucidum* as monotherapy lowered C-peptide significantly (*P* < 0.001), leaving HDL-c parameter unchanged.

As shown in Table 3, the level of oxidative stress in diabetic rats was improved with the reduction of MDA and the increase of SOD and GSH-Px following GLSP intervention. Meanwhile, The GLSP intervention also led to a reduction of ROS level compared to the model control, although the difference was not significant. Considering that MDA is produced from ROS, the higher level of MDA may promote polyunsaturated fatty acid peroxidation. Previous studies have also shown a strong relationship between MDA levels and different pathological stages of diabetes, because MDA concentration increased considerably in diabetes mellitus [21]. Thus, the controlling of MDA concentration would be helpful in maintaining a suitable level of oxidative stress. Antioxidant enzymes, including SOD and GSH-Px are vital defenses against ROS and they are important in inhibiting oxygen radical formation and usually act as biomarkers for indicating ROS production. This study found that the activities of antioxidant enzymes SOD and GSH-Px were significantly decreased in the model control group compared to the normal controls, indicating a lower antioxidant defense caused by diabetes. However, the administration of GLSP significantly enhanced the activity of SOD and GSH-Px (Table 3), suggesting that the antioxidant compounds present in GLSP might enhance plasma antioxidant capacity in diabetic rats. One plausible mechanism for interpreting the anti-hyperglycemic function of GLSP might be through its scavenging ability to protect pancreatic cells from oxygen-radical damage, supporting an increased secretion of insulin. Previous research has also demonstrated that a high dosage of antioxidant compounds had a favorable effect on glucose homeostasis in obese subjects with the improvements in their Homeostasis Model Assessment (HOMA) index, thus exerting a positive effect on insulin sensitivity [22].

Previous reports have shown the anti-hyperglycemic activity of GLSP [17, 18] and this function was further highlighted in this study (Table 1). A significant elevation of insulin levels in rats was also demonstrated after the administration of GLSP (unpublished data), which was also accompanied by a decreased blood glucose level. The current study is consistent with previously reported work [23]. Thus, the current results could further support the evidence from previous studies on the anti-hyperglycemic effect of GLSP through decreased glucose level in animals accompanied with increased insulin levels and improvements in pancreatic cell function [24–27]. However, the mechanisms associated with this benefit are not completely clear. Therefore, in light of our current results, it might be hypothesized that the benefits derived from GLSP intervention in the diabetic rats could be partially associated with its roles in promoting glycogen synthesis although gluconeogenesis was not significantly affected (*PECPK* and *G6PC1* in Fig. 1). Another plausible reason may be associated with insulin regulation. The expression level of insulin induced genes, in particular *Insig-1*, was enhanced significantly in GLSP intervention group compared to model control group. It has been shown that *Insig-1* and *Insig-2* play an important role in glucose homeostasis [28] and they also have a key function in the regulation of intracellular cholesterol and fat metabolism [29]. Consistently, previous reports have confirmed that a lower glucose concentration could promote the expression levels of *Insig-1* and *Insig-2* genes [30] and *Insig-1* inhibited lipid accumulation and free fatty acid (FFA) synthesis in a time-dependent manner [31]. In addition, the hepatic over-expression of *Insig-1* (or *Insig-2*) would also inhibit the activation of *SREBP-1*c in the rat liver [32–34], a key factor in the control of hepatic glucose metabolism and the manipulation of glucose homeostasis associated with insulin. Although there was no significant change in the expression level of *SREBP-1* with the increased expression level of *Insig* genes in our study, post transcriptional regulation might play some roles as well.

**Fig. 3:**
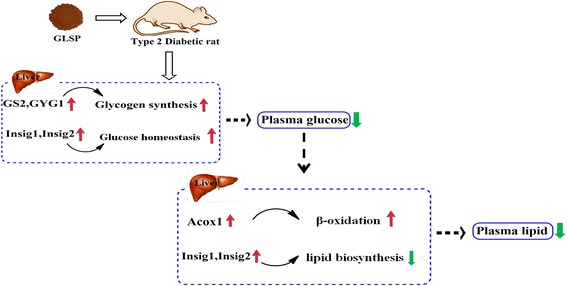
Gene regulation in metabolic pathways manipulated by GLSP in the diet of type 2 diabetic rats. Arrows in red and green indicate gene up-regulation or down-regulation, respectively. GLSP intervention reduced blood glucose level through increasing glycogen synthesis (*GS2* and *GYG1*) and glucose homeostasis (*Insig-1* and *Insig-2*). Concomitantly, the reduction of plasma lipids occurred following GLSP administration, which might be associated with the increasing of genes expression of *Acox1* and *Insig-1/2*

In this study, the significant improvement of blood lipid compositions confirmed that GLSP could be used as an efficient therapy for treating hyperlipidemia caused by diabetes and these improvements in blood cholesterol and triglyceride levels might be related to the reduced blood glucose level in the diabetic rats [35, 36]. Nevertheless, other study also suggested that *G. lucidum* consumption could result in a great suppression of elevated total cholesterol levels, which might be associated with the inhibition of the hepatic phosphoenolpyruvate carboxykinase gene expression [17]. The HMG CoA reductase activity associated with lipoprotein metabolism may also contribute to the hypolipidemic effects following GLSP treatment, which is similar to the effects of other plant medicines [37, 38]. Since *Insig* genes are also associated with lipid metabolism, acyl-CoA oxidase plays an important role in plasma lipid compositions. Fatty acid degradation in most organisms occurs primarily via β-oxidation. Both mitochondrial and peroxisomal β-oxidation catalyze the shortening of acyl-CoA esters chains, which requires the participation of β-oxidation enzymes, including acyl-CoA dehydrogenase and acyl-CoA oxidase (*Acox*) [39]. In our study, the *Acox1* gene was up-regulated by over 5-folds in the diabetic rats following GLSP treatment, which might provide some clear evidences that GLSP could reduce TG level mainly through promoting lipid β-oxidation (Fig. 2). Thus, based on above results in this study, a relationship between GLSP intervention and the expression of genes involved in glucose and lipid metabolisms in the diabetic rats is summarized in Fig. 3. In brief, GLSP in the diet of type 2 diabetic rats could reduce the blood glucose level possibly through the increasing of the gene expression level of glycogen synthesis (*GS2* and *GYG1*) and glucose homeostasis (*Insig-1* and *Insig-2*). In particular, plasma lipid compositions (TG and TC) decreased concomitantly with increased gene expression levels of *Acox1* and *Insig-1/2*.

## Conclusion

In summary, this study has shown that *G. lucidum* spores could be used as an ingredient for attenuating diabetic mellitus through potential anti-hyperglycemic and anti-hyperlipidemic activities. More importantly, the relationships between GLSP intervention and the changes in the gene expression levels of the related glucose and lipid metabolic pathways might highlight a potential model for interpreting the action of GLSP treatment.

## Materials and methods

### Materials

*G. lucidum* spores powder (CLSP) with shell-broken rate >99.9 % was provided by Chongqing Biotechnology Institute (Chongqing, China).

### Animals and diets

Twenty four healthy male Sprague–Dawley rats of ~200 g weight were purchased from the Animal Resource Centre, Medical College of PLA Military Science (Beijing, China). They were housed in wire-bottomed cages in a room with controlled temperature (23 °C) and lighting (a 12-h-light/-darkcycle) and allowed free access to food and water. The rats were randomly assigned to 3 groups (*n* = 8/group): normal control, model control and *Ganoderma lucidum* spores powder (GLSP) treatment. The rats were fed with a basic diet. After 1 week’s adaptive feeding with the basic diet, the rats were fasted for 12 h, followed by a single intravenous injection of 45 mg/ kgb.w STZ except the rats in normal control group. After 72 h injection of STZ, the blood glucose level was higher than 16.7 mmol/L demonstrating a successful induction of diabetes. Normal control and model control animals were fed with the basal diet for 4 weeks without any intervention. In contrast, GLSP was administered for the third group by oral gavages, using a feeding needle with 1 g per day for 4 consecutive weeks before they were sacrificed for the analysis. There were no casualties or obvious signs of toxicity throughout the course of the experiments and all rats involved survived. The basal diet contained 7 % fat and 13 % protein. Group food intakes and individual body weights were monitored daily throughout the study. Experimental procedures were approved by the Animal Ethics Committee of PLA Military Science and complied with the Chinese Code of Practice for the Care and Use of Animals for Scientific Purposes.

### Biochemical analysis

During the whole experiment (4 weeks), blood samples from all rats were collected from the tail vein (once a week) for blood glucose level analysis. At the end of the experiments, blood samples were collected from the femoral artery before animals were sacrificed by cervical dislocation. Blood collected was stored at −80 °C prior to chemical analyses. High-density lipoprotein-cholesterol (HDL-c) (North Kangtai Clinical Reagent Co., Beijing, China, F003-2), total cholesterol (TC) (Dong’ou Diagnosis Products Co Ltd, Zhejiang, China, F002-1) and triglyceride (TG) (Dong’ou Diagnosis Products Co Ltd, Zhejiang, China, F001-1) concentrations were measured according to the instructions of their corresponding kits, respectively. Plasma glutathione peroxidase (GSH-Px) (Jiancheng Biological Engineering Institute, Nanjing, China, A061-1), reactive oxygen species (ROS) (Sigma-Aldrich, USA, DCFH-DA, D6883) and superoxide dismutase (SOD) were measured by enzyme-linked immunosorbent assay (ELISA) (Jiancheng Biological Engineering Institute, Nanjing, China, A015). Commercially available ELISA kit was used to determine blood insulin levels (Beinglay Biotech Co., Ltd, Wuhan, China, DRE20732).

Following the cervical dislocation, the rats were dissected immediately with sterile scissors and the liver was removed, weighed and immediately frozen in liquid nitrogen and stored at −80 °C prior to RNA extraction.

### Analysis of gene expression associated with lipid and glucose metabolisms

Immediately after sacrifice, livers of all rats were quickly removed, chilled with liquid nitrogen and stored at −80 °C before homogenization for total RNA extraction using the Trizol reagent (Takara). Total RNA isolated from liver was then treated with RNase-free DNase to remove any contaminating genomic DNA and the quality and integrity of RNA were assessed using agarose gel electrophoresis stained with ethidium bromide. For RT-PCR analysis, first strand cDNA was synthesized using the PrimeScript RT reagent kit with gDNA Eraser (Takara) according to the manufacturer’s instructions. PCR was carried out in a 20 μL volume reaction containing 2 μM of each primer, 40 ng of cDNA and 10 μL of SYBR Primix ExTag. Thermal cycling conditions included an initial denaturation step at 95 °C for 5 min and then 40 cycles of 95 °C for 30 s, 58–60 °C for 30 s and 72 °C for 30 s. Fluorescence was measured at the end of each cycle. The 18S rRNA gene was used as an internal control to normalize target genes expression. Three replicates of each reaction were performed and the relative transcript quantity was calculated according to the method of 2-^ΔΔCT^ [40, 41]. Primer sequences are shown in Table 4.

**Table 4:** The primer of genes

Gene	Accession number	Forward primes(5′–3′)	Reverse primes(5′–3′)	Amplicon product (bp)	Annealing temperature (°C)
18S rRNA	NR_046237	AAACGGCTACCACATCCAAG	TTGCCCTCCAATGGATCCT	159	60
ACC	NM_022193	CAACCACTACGGCATGACTCA	CGCAGAAGCAGCCCATTACTT	155	60
FAS	NM_017332	TGCTCCCAGCTGCAG	GCCCGGTAGCTCTGGGTGTA	107	60
Acox1	NM_017340	CAAGGAGAGTGCTACGGGTTA	TTCAGGTAGCCGTTATCCAT	137	58
SREBP-1	XM_213329	GCAAGGCCATCGACTACATC	TTTCATGCCCTCCATAGACAC	161	60
Insig1	NM_022392	TTGTCGGCTTATTGTATCCCT	GCACATTATTGGCGAAATCT	147	55
Insig2	NM_178091	GGCGGAAGGAGAGACGGAGTC	AAGCCAGGAACACGCCAATGA	128	60
Acly	NM_016987	GCAGACCAGAAGGGCGTGAC	CACACTGCCTGGGCGATACAG	135	64
Fads1	NM_053445	GTTTGTGTGGGTGACGCAGAT	TTGAAGGCTGACTGGTGAACG	117	60
Gpam	NM_017274	CCTGTGGGCATCTCGTATGAT	TTCCGCAGCATTCTGATAAC	122	60
Dgat1	NM_053437	CAGATGGGGCTGCTGCTACAT	GGCGGCACCACAGATTGACAT	175	60
GS2	NM_013089	GACACTGAGCAGGGCTTTTCC	GAGGAGGGCCTGGGATACTT	90	60
GYG1	NM_031043.2	TCGCCAGCCCACAGGTT	CACCACTGTCCAAGACATCTACCA	90	60
PEPCK	NM_198780	GAAAGTTGAATGTGTGGGTGAT	TTCTGGGTTGATGGCCCTTA	79	57
G6PC1	NM_013098	GTATGGATTCCGGTGCTT	AATGCCTGACAAGACTCCA	132	55

### Statistical analysis

Results were expressed as means ± SD with SPSS software (version 13.0). The data were analyzed statistically using one-way ANOVA, repeated measures ANOVA and Tukey test (multiple comparisons). A value of *P* < 0.05 was considered as statistically significant.

## Additional file

Additional file 1: Table S1. Effect of GLSP intervention on body mass of type 2 diabetic rats. Model control: STZ induced diabetic rats without intervention; Normal control: healthy rats without intervention; GLSP:STZ induced diabetic rats with GLSP intervention.
