# Supplementary material for: Effect of Ganoderma lucidum spores intervention on glucose and lipid metabolism gene expression profiles in type 2 diabetic rats
Source: Lipids Health Dis. 2015 May 22;14:49. doi: 10.1186/s12944-015-0045-y (PMC4443549; doi:10.1186/s12944-015-0045-y)
Supplement: Additional file 1: Table S1. — Effect of GLSP intervention on body mass of type 2 diabetic rats. Model control: STZ induced diabetic rats without intervention; Normal control: healthy rats without intervention; GLSP:STZ induced diabetic rats with GLSP intervention. [file 12944_2015_45_MOESM1_ESM.doc]

**Supplementary File**

**Table 1 Effect of GLSP intervention on body mass of type 2 diabetic rats**

| Body mass (g) | | | | | | | RMANOVA  F value | P  value |
| --- | --- | --- | --- | --- | --- | --- | --- | --- |
| Group | Initial weight | 72h | 1 week | 2 week | 3 week | 4 week |
| Normal control | 206.20±7.19^a^ | 216.80±6.61^a^ | 235.00±3.32^a^ | 245.40±3.97^a^ | 249.00±3.32^a^ | 260.00±5.15^a^ | 108.72 | <0.001^**^ |
| Model control | 204.60±7.83^a^ | 201.20±9.42^b^ | 202.60±9.32^b^ | 181.20±14.25^b^ | 216.20±13.07^b^ | 204.60±18.24^b^ | 26.39 | <0.001^**^ |
| GLSP intervention | 202.60±8.03^a^ | 203.30±7.26^b^ | 199.00±6.58^b^ | 201.30±15.45^b^ | 212.50±12.86^b^ | 220.80±14.60^b^ | 16.64 | <0.001^**^ |

Denote: Results are expressed as means ± SD (n﹦8, one-way ANOVA). Different superscript lowercase letters on the table indicate significant difference (P<0.05). RMANOVA: Repeated measures ANOVA. **Strongly significant (P value: p<0.001). Normal control: healthy rats without intervention; Model control: STZ induced diabetic rats without intervention; GLSP intervention：STZ induced diabetic rats with GLSP intervention.
